# Artificial Intelligence for Liquid Biopsy: FTIR Spectroscopy and Autoencoder-Based Detection of Cancer Biomarkers in Extracellular Vesicles

**DOI:** 10.3390/cells14231909

**Published:** 2025-12-02

**Authors:** Riccardo Di Santo, Benedetta Niccolini, Enrico Rosa, Marco De Spirito, Fabrizio Pizzolante, Dario Pitocco, Linda Tartaglione, Alessandro Rizzi, Umberto Basile, Valentina Petito, Antonio Gasbarrini, Guido Gigante, Gabriele Ciasca

**Affiliations:** 1 Department of Life Science, Health and Health Professions, Link Campus University, 00165 Rome, Italy; 2 Dipartimento di Neuroscienze, Sezione di Fisica, Università Cattolica del Sacro Cuore (UCSC), Largo Francesco Vito 1, 00168 Rome, Italy; 3 Dipartimento di Scienze della Vita e Sanità Pubblica, Sezione di Medicina Genomica, Università Cattolica del Sacro Cuore (UCSC), Largo Francesco Vito 1, 00168 Rome, Italy; 4 Unità Operativa Complessa Fisica per le Scienze della Vita, Dipartimento di Diagnostica per Immagini, Radioterapia Oncologica ed Ematologia, Fondazione Policlinico Universitario Agostino Gemelli IRCCS, 00168 Rome, Italy; 5 CEMAD, Medical and Surgery Sciences Department, Fondazione Policlinico Universitario Agostino Gemelli IRCCS, 00168 Rome, Italy; fabrizio.pizzolante@policlinicogemelli.it; 6 UOSA Diabetologia, Fondazione Policlinico Universitario Agostino Gemelli IRCCS, 00168 Rome, Italy; 7 Department of Clinical Pathology, Santa Maria Goretti Hospital, 04100 Latina, Italy; u.basile@ausl.latina.it; 8 CeMAD Translational Research Laboratories Digestive Disease Center, Department of Medical and Surgical Sciences, Fondazione Policlinico Universitario Agostino Gemelli IRCCS, 00168 Rome, Italy; 9 Dipartimento di Medicina e Chirurgia Traslazionale, Università Cattolica del Sacro Cuore (UCSC), 00168 Rome, Italy; 10 Italian National Institute of Health, National Center for Radiation Protection and Computational Physics, viale Regina Elena 299, 00161 Rome, Italy

**Keywords:** extracellular vesicles, FTIR spectroscopy, autoencoder, latent features, liquid biopsy, hepatocellular carcinoma, machine learning, cancer biomarkers, infrared spectroscopy

## Abstract

Extracellular vesicles (EVs) are increasingly recognized as promising non-invasive biomarkers for cancer and other diseases, but their clinical translation remains limited by the lack of comprehensive characterization strategies. Spectroscopic approaches such as Fourier-transform infrared (FTIR) spectroscopy can provide a global biochemical fingerprint of intact EVs, but their interpretation requires advanced analytical tools. In this study, we applied an autoencoder-based framework to attenuated total reflection FTIR (ATR-FTIR) spectra of blood-derived components, including plasma, red blood cells (RBCs), RBC-ghosts, and EVs, comprising 278 samples collected from 135 patients, to obtain latent features capable of capturing biologically meaningful variability. The autoencoder compressed spectra into 12 latent features while preserving spectral information with low reconstruction error. Unsupervised UMAP projection of the latent features separated the blood components into different clusters, supporting their biological relevance. The model was then applied to EV spectra from patients with hepatocellular carcinoma (HCC) and cirrhotic controls. Four features significantly differed between the two groups, and an elastic-net regularized logistic model evaluated with a leave-one-out cross-validation framework retained a single latent feature, achieving an out-of-fold ROC AUC of 0.785 (95% CI 0.602–0.967), with performance broadly comparable to that typically reported for AFP, the most commonly used biomarker for HCC. This study provides the first proof-of-concept that an autoencoder can be applied to FTIR spectra of EVs, extracting biologically relevant latent features with potential application in cancer detection.

## 1. Introduction

Virtually all cells continuously release small lipid-based extracellular vesicles (EVs) into the extracellular milieu, enabling communication with neighbouring cells. Since their molecular cargo reflects the physiological or pathological state of the originating cells, EVs hold great promise as non-invasive biomarkers for a wide range of human diseases, including cancer as well as neurological, cardiovascular and metabolic disorders [1,2,3,4,5,6]. Despite the increasing clinical interest in EVs, several challenges continue to limit their widespread application in diagnostic and prognostic settings [7,8]. EVs represent a highly complex and heterogeneous family of vesicles; although they are abundantly released into human biofluids and are therefore easily accessible, their specific isolation remains technically challenging [9]. Conventional EV characterization techniques, particularly immunochemical methods such as ELISA and Western blotting, typically focus on single or a few EV antigens. This limited scope can compromise diagnostic accuracy, which explains why most EV-based diagnostic assays under development increasingly adopt multi-marker EV panels [10,11].

In this context, there is a strong need for novel approaches enabling comprehensive EV characterization. Fourier-transform infrared (FTIR) spectroscopy offers a global biochemical fingerprint of intact vesicles, simultaneously capturing their lipid, protein, and nucleic acid content [12,13,14,15,16,17]. Moreover, this technique is rapid, non-invasive, label-free, and cost-effective. However, FTIR spectra of complex biological systems, such as EVs, are inherently high-dimensional and challenging to interpret reliably. To overcome these limitations, machine learning approaches have been increasingly employed to reduce data dimensionality and to reveal spectral features that may carry biological relevance [15,18,19,20,21].

In this study, we applied autoencoders to identify latent spectral features with diagnostic potential in the FTIR spectra of EVs isolated from cancer patients. Autoencoders are unsupervised neural networks designed to compress high-dimensional data into a reduced set of latent features, while retaining the essential information needed for data reconstruction [22,23]. Previous studies have successfully employed autoencoders for the analysis of FTIR spectra in various applications, supporting their potential for feature extraction and dimensionality reduction [24,25,26]. However, to the best of our knowledge, autoencoders have not yet been applied to the FTIR analysis of EVs, nor evaluated for their diagnostic potential in patient-derived samples. Specifically, we trained our model on FTIR spectra from plasma, red blood cells (RBCs), and EVs, using 278 biological samples derived from 135 patients, to establish a latent space capable of reconstructing spectral data independently of their biological origin. Training on multiple blood components rather than EVs alone provided stronger validation of the model and increased the robustness of the latent features. We then applied the trained model to EVs obtained from hepatocellular carcinoma (HCC) patients and cirrhotic controls, evaluating the diagnostic potential of the latent spectral features extracted by the autoencoder. HCC remains a leading cause of cancer-related mortality, and current AASLD/EASL guidelines recommend semi-annual surveillance in patients with cirrhosis, typically using ultrasound with or without serum alpha-fetoprotein (AFP) [27,28]. However, the sensitivity of these tools remains suboptimal, particularly in early-stage disease, highlighting the need for novel, minimally invasive biomarkers.

## 2. Materials and Methods

### 2.1. Sample Collection and Preparation

A total of 278 biological samples were obtained from 135 patients recruited at the Fondazione Policlinico Gemelli (FPG) IRCCS Hospital (Rome, Italy). All procedures were approved by the local institutional ethics committee (Protocol numbers: 2078; 5452; 0027695/23) and conducted in accordance with the Declaration of Helsinki. Written informed consent was obtained from all participants. The majority of patients (*n* = 71) were affected by type 1 diabetes mellitus (T1DM), a recognized risk factor for several pathological conditions including hepatocellular carcinoma (HCC). Eligible participants were adults (≥18 years) with a diagnosis of T1DM according to ADA/WHO criteria, disease duration ≥ 6 months, and stable therapy for ≥3 months. Exclusion criteria were age < 18 years, pregnancy, or concomitant chronic inflammatory demyelinating polyradiculoneuropathy, systemic vasculitis, autoimmune gastritis, or unstable thyroid disease. Thirty-eight healthy donors were also included in this study.

In addition, 9 patients with non-viral hepatocellular carcinoma (HCC) and 16 patients with cirrhosis of metabolic origin were enrolled. Patients with newly diagnosed HCC were recruited according to EASL criteria. Liver function, evaluated using Child–Pugh scores, was comparable between groups (median 7.5 [IQR 5.0–9.75] in HCC and 9.0 [IQR 8.0–10.06] in cirrhosis; *p* > 0.5). Cirrhosis was confirmed by clinical, radiological, or histological evidence in patients free from HCC or other malignancies.

Whole blood was processed to obtain plasma, red blood cells (RBCs), RBC-ghosts (RBC-G), and extracellular vesicles (EVs). Plasma was separated by centrifugation at 3000× *g* for 10 min by using Labnet Hermle Z300 (Labnet International, Edison, NJ, USA) and collected for ATR-FTIR analysis. RBCs were washed twice (1000× *g*, 2 min), and 10 µL of the pellet were resuspended in 1.5 mL 0.9% NaCl to obtain a final suspension for spectral measurements. RBC-G were prepared by hypotonic lysis: 150 µL of RBC pellet were dispersed in 1 mL ultrapure water (Milli-Q water purification system, Millipore, Burlington, MA, USA), gently agitated for 15 min at room temperature, and centrifuged at 13,000× *g* for 15 min. The pellet was washed repeatedly (3000× *g*, 10 min) until a clear supernatant was obtained.

EVs were isolated from plasma either by ultracentrifugation or by precipitation. For ultracentrifugation, 2 mL of plasma underwent sequential centrifugation (1500× *g*, 30 min; 3200× *g*, 30 min) with pellet removal. The supernatant was clarified (11,000× *g*, 30 min, 4 °C; Optima XPN-100, Beckman Coulter, Krefeld, Germany), followed by two ultracentrifugations (100,000× *g*, 2 h, 4 °C) to pellet EVs, which were resuspended in 100 µL ultrapure water. For precipitation, EVs were isolated using the ExoQuick ULTRA kit (System Biosciences, Palo Alto, CA, USA) according to the manufacturer’s instructions. Briefly, clarified plasma was obtained by centrifugation (3000× *g*, 15 min; 12,000× *g*, 10 min), incubated with ExoQuick reagent (System Biosciences, Palo Alto, CA, USA) - 67 µL per 250 µL plasma - for 30 min, centrifuged, and resuspended in kit buffer. Preparations were further purified on kit-provided columns to obtain high-purity EVs.

### 2.2. ATR-FTIR Spectral Acquisition and Pre-Processing

Spectroscopic measurements were performed using a Bruker Alpha II FTIR spectrometer (Bruker Corporation, Billerica, MA, USA) equipped with an Eco-ATR. A 20 μL aliquot of each sample was deposited onto a Diamond/ZnSe crystal and allowed to air-dry prior to analysis. Spectra were acquired in the 4000–1000 cm^−1^ range, averaging 24 scans at a resolution of 2 cm^−1^. A background spectrum was recorded in air before each measurement and automatically subtracted from the corresponding sample spectrum. The ATR crystal was thoroughly cleaned with ethanol followed by distilled water between acquisitions to avoid cross-contamination. Spectra were recorded in absorbance units using OPUS 8.5 SP1 software (Bruker Corporation, Billerica, MA, USA). Atmospheric contributions from CO_2_ and water vapor were corrected prior to export using the built-in atmospheric compensation routines provided in OPUS 8.5 SP1.

Following acquisition, all spectra underwent a standardized pre-processing pipeline. Baseline distortions were corrected by subtracting a linear fit calculated within the flat region of the spectra between 3900 and 4000 cm^−1^. Each spectrum was then normalized to a 0–1 range to account for intensity variations across samples. Finally, spectral binning was performed within the 4000–1000 cm^−1^ region using a bin width of 8, reducing the dimensionality from 1460 to 375 variables. This pre-processing workflow ensured the generation of comparable and noise-reduced spectra suitable for subsequent machine learning analysis.

### 2.3. Autoencoder Modeling and Statistical Analysis

An autoencoder neural network was implemented in R v4.4.0 [29] using the keras3 package [29]. Each spectrum (375 variables after pre-processing) was used as input. The encoder consisted of three fully connected layers (128, 64, and 32 units) leading to a latent space of 12 neurons, while the decoder mirrored this architecture to reconstruct the original spectra. All hidden layers used LeakyReLU activation functions with He-normal initialization. To reduce overfitting, we applied dropout (0.1) and L2 regularization (λ = 1 × 10^−4^) to all dense layers except the output. The network was trained to minimize the mean absolute error (MAE) between original and reconstructed spectra using the Adam optimizer (learning rate = 1 × 10^−4^), with a batch size of 32 and early stopping. Model performance was assessed through convergence of the loss function and by calculating the root mean square error (RMSE) between original and reconstructed spectra.

The 12 latent features extracted from the encoder were used for unsupervised clustering and statistical analysis. UMAP was applied for dimensionality reduction and visualization using the umap R package v0.2.10.0 [30]. Welch’s *t*-test was performed with base R functions, and *p*-values were adjusted for multiple testing with the Benjamini–Hochberg procedure.

For classification of HCC and cirrhotic patients (*n* = 25) we used elastic-net logistic regression (glmnet) [31] (mixing parameter α = 0.66). Model selection and evaluation were conducted with leave-one-out cross-validation (LOOCV) and applying the λ.1se rule to favor parsimony, given the limited sample size. Out-of-fold decision scores at λ.1se were used to compute ROC and AUC with pROC [32]; The optimal operating point was defined by the Youden index, from which TP/FP/TN/FN and accuracy were derived. Boxplots and descriptive summaries were generated using the ggplot2 v4.0.1 and gtsummary v2.4.0 packages [33]. 

## 3. Results

### 3.1. Model Design and Validation on Blood-Derived Components

**Figure 1:**
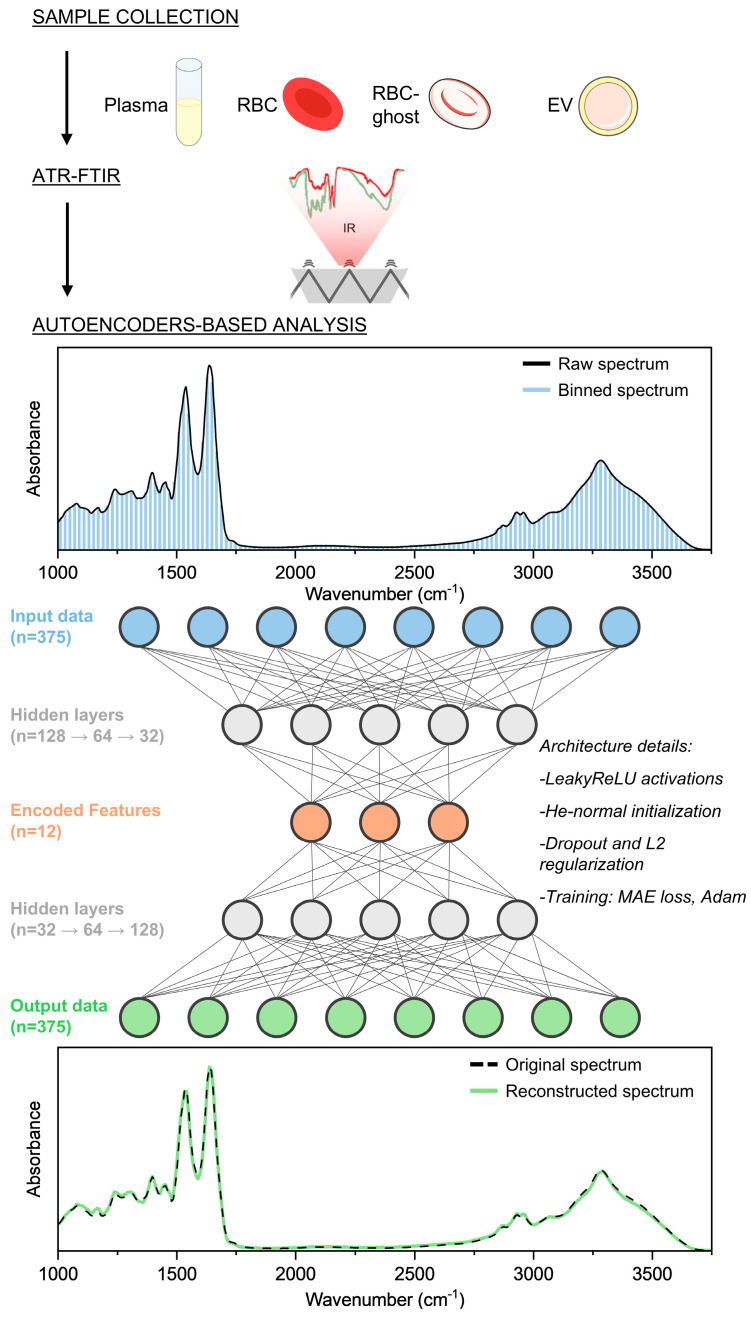
Schematic representation of the experimental pipeline, including sample collection, ATR-FTIR spectral acquisition, and autoencoder-based analysis. FTIR spectra were binned to 375 variables (input layer), compressed through multiple hidden layers into a 12-dimensional latent space, and then reconstructed back to the original dimensionality.

We first developed an autoencoder-based framework to analyze FTIR spectra from blood-derived components. The workflow included sample collection, acquisition of spectral data, and dimensionality reduction through the autoencoder architecture (Figure 1). Spectral data from biological samples, including plasma, red blood cells (RBCs), RBC-ghosts (RBC-G), and extracellular vesicles (EVs), were acquired using Fourier-transform infrared (FTIR) spectroscopy in attenuated total reflection mode (ATR-FTIR). This technique provides vibrational spectra of biological samples, capturing their global biochemical composition and molecular fingerprints. Each spectrum underwent a pre-processing pipeline comprising baseline correction, normalization, and binning, to standardize the data and avoid artefacts, as detailed in Section 2.

We implemented a feed-forward autoencoder to compress FTIR spectra into a reduced latent space and subsequently reconstruct the input data. Spectral profiles were represented by 375 input variables after binning, which were compressed into a 12-dimensional latent space before being reconstructed back to the original dimensionality (375 variables).

Reconstruction accuracy was assessed through the loss function, which converged to a mean absolute error (MAE) of 0.044, and by calculating the root mean square error (RMSE; average RMSE = 0.032 ± 0.022), confirming that the autoencoder latent space effectively preserved the essential spectral information required for accurate FTIR reconstruction. Each spectrum could be reduced to 12 latent features and accurately reconstructed. 

**Figure 2:**
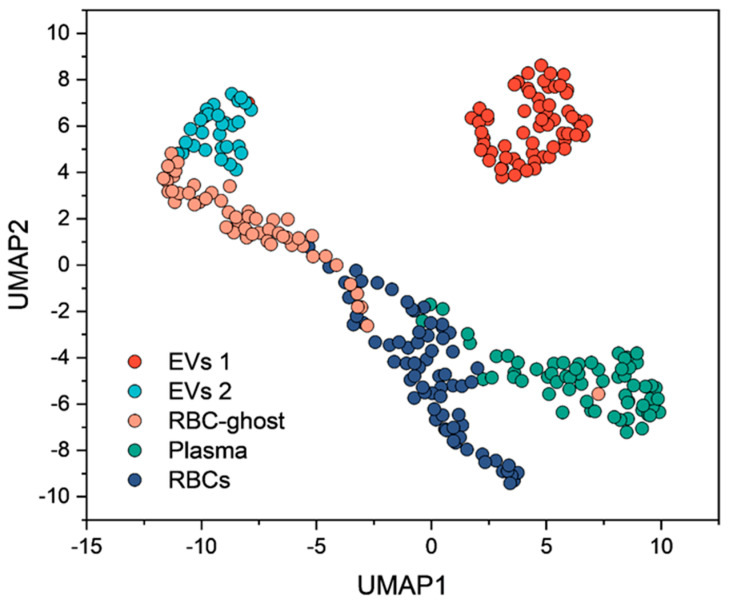
Latent features extracted by the autoencoder were projected into two dimensions using UMAP. Samples clustered according to their biological origin, with RBCs, plasma, RBC-ghosts, and EVs forming distinct groups. Notably, EVs segregated into two clusters: EVs1, isolated from plasma using a precipitation-based kit, and EVs2, isolated by ultracentrifugation. This separation suggests heterogeneity within the vesicle population depending on the isolation method.

We then evaluated whether these latent features also captured biological meaning by distinguishing different blood-derived components. To this end, we applied Uniform Manifold Approximation and Projection (UMAP), an unsupervised dimensionality reduction method that projects high-dimensional data into a lower-dimensional space while preserving both local and global structure. Figure 2 shows the UMAP projection of all biological samples analyzed in this study, where the 12 latent features extracted by the autoencoder were further reduced to two variables (UMAP1 and UMAP2). Interestingly, samples belonging to different blood-derived components formed distinct clusters: plasma (green), RBCs (dark blue), and RBC-ghosts (pink) each grouped separately, while EVs segregated into two clusters corresponding to different isolation methods, EVs1 obtained by precipitation-based kit (red) and EVs2 obtained by ultracentrifugation (light blue). This clustering emerged in a completely unsupervised manner, without the UMAP algorithm receiving any information about sample labels, indicating that the autoencoder-derived features retained biologically meaningful variability across sample types.

### 3.2. Evaluation of EV Spectral Latent Features for Cancer Detection

Latent features extracted by the autoencoder were shown to preserve biological relevance by distinguishing among different blood-derived components (Figure 2). We next evaluated whether these features could also have potential clinical utility. To this end, we obtained ATR-FTIR spectra of EVs isolated from patients with hepatocellular carcinoma (HCC) and from cirrhotic patients used as controls, since cirrhosis represents the major risk factor for HCC. EV spectra were pre-processed, and the previously trained model was applied to extract latent features and assess their ability to discriminate between pathological and control groups.

**Figure 3:**
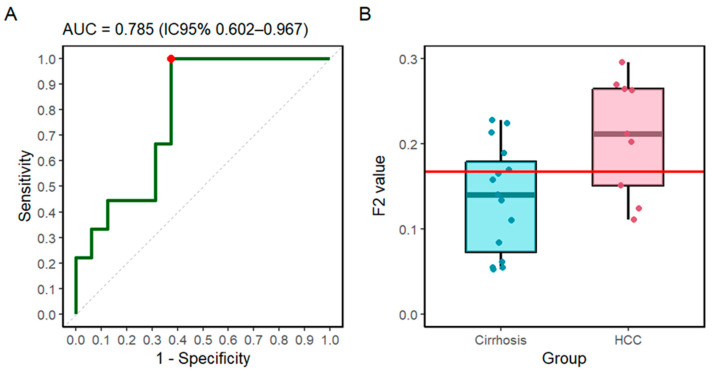
(**A**) ROC curve from leave-one-out out-of-fold scores of the elastic-net model (λ.1se); AUC = 0.785 (95% CI 0.602–0.967). The red dot marks the Youden point. (**B**) Box plot of the F2 latent feature by group (cirrhosis vs. HCC); the horizontal red line shows the Youden cut-off.

Table 1 reports the 12 latent features extracted from EV spectra of HCC and cirrhotic patients. Features are ranked from top to bottom according to their statistical significance in differentiating the two groups. Significance was assessed using Welch’s *t*-test, and *p*-values were corrected for multiple testing with the Benjamini–Hochberg false discovery rate (FDR), reported as *q*-values. As expected with a limited number of features (*n* = 12), several *q*-values resulted in identical adjusted values Four latent features (F2, F5, F10, and F11) reached statistical significance after correction (*q*-value = 0.041). Beyond testing for group differences, we evaluated the classification performance of the latent features. We used a regularized strategy restricted to the four features that remained significant after FDR correction, fitting an elastic-net logistic model and assessing it under a leave-one-out cross-validation (LOOCV) framework. To favor parsimony, we report the λ.1se solution. The λ.1se model retained a single latent feature (F2), consistent with the need to limit model complexity given the small sample size. The out-of-fold ROC (Figure 3A) yielded an AUC of 0.785 (95% CI 0.602–0.967), indicating a fair ability to discriminate HCC from cirrhosis. A permutation test assessing whether this value could arise by chance is reported in Supplementary Figure S1. For context, AFP measured within the same cohort yielded a comparable diagnostic performance (AUC = 0.75; 95% CI 0.53–0.97). This supports the view that the latent feature F2 captures clinically relevant information at a level consistent with an established biomarker. The distribution of F2 values in the two groups is shown in Figure 3B (box plot), confirming higher levels in HCC compared with cirrhosis; the horizontal red line marks the Youden cut-off derived from the ROC analysis.

**Figure 4:**
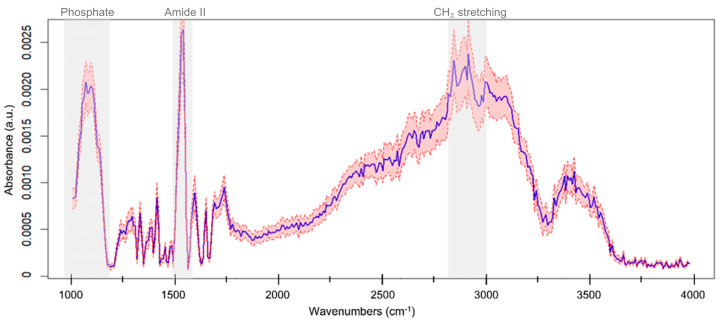
For each subject (*n* = 25; cirrhosis or HCC), the latent feature F2 was increased by 5% while keeping the remaining features fixed; the modified latent vector was decoded to the spectral domain and the differential spectrum was computed as (original − modified). The solid line shows the mean differential spectrum across subjects and the shaded envelope the 95% confidence band. Grey boxes highlight key biochemical regions showing consistent changes: ~1010–1015 cm^−1^ (sugar/phosphate modes of nucleic acids), Amide II ~1540–1560 cm^−1^, and CH_2_ lipid stretching ~2850–2920 cm^−1^.

As a stability check on variable selection, we ran stepwise AIC logistic regression; the resulting model converged on the same latent feature (F2). With F2 confirmed as stable, we then probed its biomolecular meaning via a latent-space sensitivity analysis. Specifically, we increased F2 by a small fixed amount (0.01, approximately 5% of its range across the dataset) while keeping all other latent features unchanged, decoded the perturbed latent vector to obtain the corresponding reconstructed spectrum, and computed the differential spectrum as the difference between the perturbed and unperturbed reconstructions. This procedure was repeated for all 25 subjects (cirrhosis and HCC), and the resulting differential spectra were averaged to generate a mean profile with 95% confidence bands (Figure 4). The resulting profile shows consistent changes in spectroscopic regions with clear biochemical attribution [17]: a peak around 1100–1150 cm^−1^ (commonly linked to deoxyribose C–O and/or the C–O–P phosphodiester backbone of nucleic acids); a marked response within Amide II (~1540–1560 cm^−1^); and prominent variations in the CH_2_ lipid stretching region (~2850–2920 cm^−1^). These bands (highlighted in grey in Figure 4) indicate that F2 encodes information from nucleic acids, proteins, and lipids, with the largest contribution in Amide II.

## 4. Discussion

In this work, an autoencoder-based approach was applied to ATR-FTIR spectra of different biological samples, including plasma, red blood cells (RBCs), and extracellular vesicles (EVs), to reduce data dimensionality while preserving the essential spectral information (Figure 1). In this regard, our framework offers a complementary approach compared with classical FTIR machine-learning pipelines. Methods such as PCA or PLS-DA rely on linear projections and therefore capture only orthogonal components of variance, whereas autoencoders learn non-linear latent manifolds capable of encoding subtle and distributed spectral interactions [34,35]. Additionally, while supervised approaches such as PLS-DA or SVM require class labels and may overfit small biomedical cohorts, our unsupervised model extracts latent features solely from the intrinsic structure of the spectra. These characteristics make autoencoders particularly suitable for exploratory biomarker discovery in heterogeneous EV populations. Previous studies have applied autoencoders to spectroscopic data, including infrared and near-infrared spectra [24,25], primarily to enhance the analysis of spectroscopic data of complex heterogeneous systems. More recently, Jang et al. introduced a semi-supervised autoencoder for chemical gas classification using FTIR spectra, achieving superior performance compared to conventional approaches [26]. Importantly, while autoencoder-based approaches have also been explored in the context of EVs using Raman spectroscopy [36,37], to our knowledge no previous study has applied autoencoders to FTIR spectra of EVs, nor evaluated their diagnostic potential in patient-derived samples. 

In our study, the autoencoder compressed each spectrum into a 12-dimensional latent space while preserving spectral information with low reconstruction error. A key aspect of our approach was that the model was trained not only on EVs but also on other blood-derived components, including plasma and RBCs. We have also included RBC-ghosts in the training set so that the autoencoder would learn spectral features associated specifically with the membrane component, independent of cytoplasmic content. 

This strategy was designed to ensure that the latent space captured variability across different sample types, thereby increasing the robustness of the model and reducing the risk of overfitting. 

Unsupervised UMAP projection of the latent features separated plasma, RBCs, RBC-ghosts, and EVs into distinct clusters (Figure 2). Interestingly, groups with higher biological similarity were in closer proximity, with RBC-ghosts clustering near RBCs, consistent with their shared membrane composition. EVs segregated into two clusters according to the isolation method, reflecting the known variability introduced by different purification strategies [38]. EVs isolated by ultracentrifugation (EV_2_) localized closer to RBC-ghosts, suggesting a stronger membrane-related signal, whereas those obtained with precipitation kits (EV_1_) were positioned nearer to plasma. These patterns indicate that the model not only compressed spectral data efficiently but also captured biologically meaningful relationships.

We next assessed whether the latent features could capture clinically relevant variability. Previous works from our group has already demonstrated that FTIR analysis of EVs holds promise for the detection of HCC [15,16]. As such, the trained autoencoder was applied to EV spectra from patients with hepatocellular carcinoma (HCC) and cirrhotic controls, since cirrhosis represents the major risk factor for HCC [39,40].

Statistical analysis revealed four features (F2, F5, F10, and F11) that differed significantly between the two groups after multiple testing correction (Table 1). Among them, a regularized logistic regression under a leave-one-out cross-validation framework consistently selected F2 as the most informative. The resulting model achieved an AUC of 0.785, supporting the potential of this feature for disease discrimination (Figure 3). Importantly, the analysis in Figure 4 revealed that F2 encodes spectral variability associated with nucleic acids, proteins, and lipids, with the largest contribution in Amide II. This pattern aligns with prior evidence identifying Amide II as the most informative region for distinguishing HCC from cirrhosis in this cohort [16], thereby supporting both the parsimony of the final model and the biological plausibility of the selected feature.

The classification performance of the latent feature F2 is comparable to that reported for alpha-fetoprotein (AFP), the most widely used circulating biomarker for HCC. However, AFP suffers from important limitations, with approximately 30% of HCC patients being AFP-negative and overall accuracy remaining suboptimal [41,42]. To overcome such limitations, clinical practice increasingly relies on multimarker strategies to improve diagnostic accuracy [43,44]. In the case of HCC, for instance, AFP is often combined with other biomarkers, such as PIVKA-II, to enhance performance [45]. In this context, the spectral feature identified in our study, despite not achieving a very high AUC, may be ideally suited for integration into multimarker panels. Its spectroscopic nature makes it fundamentally different from conventional protein biomarkers, offering complementary information. Future studies should thus explore whether combining autoencoder-derived spectral features with established clinical biomarkers could enhance diagnostic accuracy and support their translation into clinical workflows.

Despite the potential of our findings, this study has limitations that must be acknowledged. First, the number of patients included in the clinical comparison was relatively small (9 HCC and 16 cirrhotic controls), which limits statistical power and generalizability. Second, the autoencoder model was trained and evaluated on samples derived from the same cohort, without an independent external validation set. As such, model robustness in different clinical contexts could not be assessed. Future studies should address these limitations by incorporating larger and independently collected patient cohorts to validate the diagnostic performance of autoencoder-derived FTIR features. In addition, expanding the analysis to other cancer types or pathological conditions could help to evaluate the broader applicability of autoencoder-derived FTIR features of EVs. Finally, combining autoencoder-based spectral features with complementary machine learning classifiers may further improve diagnostic accuracy and support the translation of this methodology into clinical workflows.

An additional methodological challenge for FTIR-based liquid biopsy is the lack of standardized pre-processing protocols. Recent methodological studies have shown that differences in spectral normalization, baseline correction, water subtraction, and noise handling can substantially affect diagnostic performance and limit reproducibility across laboratories [46]. Similar conclusions have been reported in broader reviews of IR liquid biopsy workflows, where subtle variations in sample drying, scattering artifacts, or normalization strategies were shown to modify the biochemical profile of the spectra and hinder cross-study comparability [47]. These observations underscore the need for harmonized and rigorously validated analytical pipelines to ensure that FTIR-based EV biomarkers can be reliably translated into clinical settings.

Beyond analytical standardization, a second key requirement for clinical translation is model interpretability. As FTIR spectral features carry biochemical meaning, ensuring that machine-learning predictions remain transparent and clinically interpretable is essential for adoption in real-world diagnostic settings. As recently emphasized in the field of medical AI, deep learning models, despite their predictive power, are often difficult to integrate into clinical practice. Explainable AI approaches aim to address this gap by providing transparency on how models derive their predictions, enabling clinicians to interpret model behaviour [48]. In FTIR-based diagnostics, where spectral features have biochemical meaning, interpretable models are essential to ensure clinical acceptability.

In this regard, a major strength of machine learning approaches is their potential to provide diagnostic outputs directly to clinicians, rather than requiring labor-intensive interpretation by specialists [49]. Once properly validated, such models could be integrated into clinical practice through straightforward platforms, making advanced data analysis accessible without the need for personnel specifically trained in spectroscopy or bioinformatics.

Another promising strategy involves the integration of plasmonic nanostructures with vibrational spectroscopy [50]. These substrates can strongly enhance the electromagnetic field at the nanoscale, enabling the detection of vibrational fingerprints of EVs directly in liquid samples and even the selective capture of specific EV subpopulations. Although this field is still developing, recent advances demonstrating the feasibility of plasmonic-enhanced EV detection [16,51].

In conclusion, this study provides the first demonstration that autoencoder-derived latent features from FTIR spectra of EVs can capture biologically meaningful information, effectively discerning between different blood components. In addition, they show potential clinical utility by discriminating EVs from cancer patients and controls, supporting their exploration as non-invasive biomarkers in future studies.

## Figures and Tables

**Table 1:** Latent features were extracted using the autoencoder model and compared between HCC (*n* = 9) and cirrhotic patients (*n* = 16). Features are ranked according to their statistical significance. Reported values include *p*-values (Welch’s *t*-test) and *q*-values (Benjamini–Hochberg FDR correction). Four features (F2, F5, F10, and F11) remained significant after correction.

Latent Features	HCC N = 9 ^1^	Cirrhosis N = 16 ^1^	*p*-Value ^2^	*q*-Value ^3^
F2	0.21 ± 0.07	0.13 ± 0.07	0.010	0.041
F5	0.10 ± 0.02	0.06 ± 0.05	0.011	0.041
F10	0.19 ± 0.03	0.12 ± 0.08	0.005	0.041
F11	0.03 ± 0.04	0.11 ± 0.11	0.014	0.041
F1	0.08 ± 0.03	0.06 ± 0.03	0.13	0.2
F3	−0.03 ± 0.01	−0.03 ± 0.01	0.8	>0.9
F4	0.01 ± 0.02	0.01 ± 0.03	>0.9	>0.9
F6	0.15 ± 0.06	0.09 ± 0.14	0.2	0.3
F7	0.04 ± 0.02	0.02 ± 0.03	0.064	0.13
F8	0.20 ± 0.10	0.27 ± 0.18	0.2	0.3
F9	−0.01 ± 0.02	−0.01 ± 0.04	>0.9	>0.9
F12	0.15 ± 0.07	0.07 ± 0.10	0.036	0.087

^1^ Mean ± SD. ^2^ Welch Two Sample *t*-test. ^3^ Benjamini & Hochberg correction for multiple testing.

## Data Availability

The raw data supporting the conclusions of this article will be made available by the authors on request.

## Supplementary Materials

The following supporting information can be downloaded at: https://www.mdpi.com/article/10.3390/cells14231909/s1, Figure S1: Right-tailed permutation test on the logistic model shown in Figure 3A.

## Abbreviations

The following abbreviations are used in this manuscript:

EVs	Extracellular Vesicles
FTIR	Fourier-Transform Infrared
ATR	Attenuated Total Reflection
HCC	Hepatocellular Carcinoma
RBC	Red Blood Cells
RBC-G	Red Blood Cell Ghosts
UMAP	Uniform Manifold Approximation and Projection
LOOCV	Leave-One-Out Cross-Validation
ROC	Receiver Operating Characteristic
AUC	Area Under the Curve
MAE	Mean Absolute Error
RMSE	Root Mean Square Error
FDR	False Discovery Rate
AFP	Alpha-Fetoprotein
ADA	American Diabetes Association
WHO	World Health Organization
EASL	European Association for the Study of the Liver
