# Supplementary material for: Artificial Intelligence for Liquid Biopsy: FTIR Spectroscopy and Autoencoder-Based Detection of Cancer Biomarkers in Extracellular Vesicles
Source: Cells. 2025 Dec 2;14(23):1909. doi: 10.3390/cells14231909 (PMC12691381; doi:10.3390/cells14231909)
Supplement: Supplementary file 1 [file cells-14-01909-s001.zip › cells-3949549-supplementary.pdf]

Supplementary Materials

Artificial Intelligence for Liquid Biopsy: FTIR Spectroscopy and Autoencoder-Based Detection of Cancer Biomarkers in Extracellular Vesicles

R. Di Santo[1, 2, *, †], B. Niccolini[2, 3, 4, †], E. Rosa[2, 4], M. De Spirito[2, 4], F. Pizzolante[5], D. Pitocco[6], L. Tartaglione[6], A. Rizzi[6], U. Basile[7], V. Petito[8, 9], A. Gasbarrini[8, 9], G. Gigante[10] and G. Ciasca[2, 4]

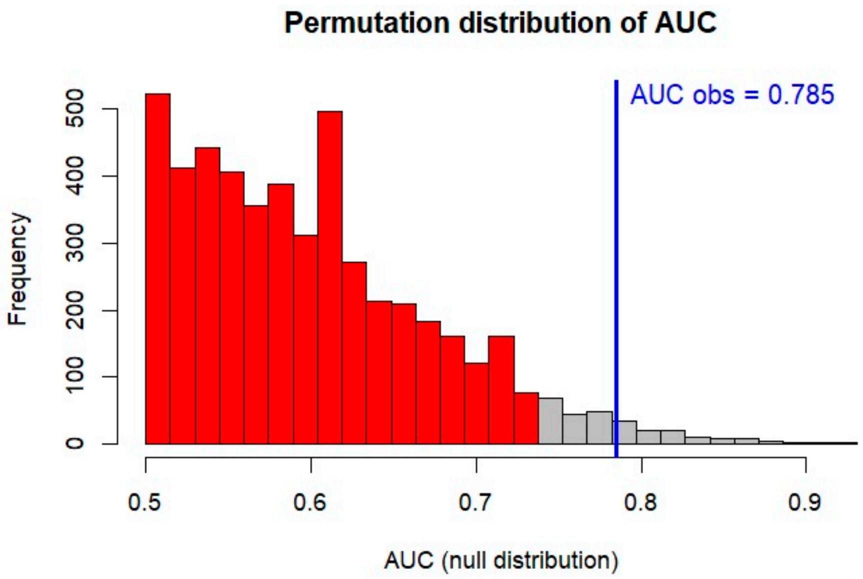

Figure S1: Right-tailed permutation test on the logistic model shown in Figure 3A. The class labels were randomly scrambled 5,000 times and the AUC was recomputed to obtain the null distribution expected under the assumption of no association between model predictions and true labels. The AUC obtained with the real labels lies above the 95th percentile of this null distribution (permutation p = 0.022), indicating that the observed performance is unlikely to arise by chance.
